# Closing the Domain Gap: Can Pseudo-Labels from Synthetic UAV Data Enable Real-World Flood Segmentation?

**DOI:** 10.3390/s25123586

**Published:** 2025-06-06

**Authors:** Georgios Simantiris, Konstantinos Bacharidis, Costas Panagiotakis

**Affiliations:** 1 Department of Management Science and Technology, Hellenic Mediterranean University, 72100 Agios Nikolaos, Greece; kbach@ics.forth.gr; 2 Institute of Computer Science, FORTH (Foundation for Research & Technology–Hellas), 70013 Heraklion, Greece

**Keywords:** unsupervised image segmentation, deep learning, pseudo-labels, flood segmentation, unmanned aerial vehicles, image inpainting, text-to-image synthesis

## Abstract

We present a novel methodology for generating and filtering synthetic Unmanned Aerial Vehicle (UAV) flood imagery to enhance the generalization capabilities of segmentation models. Our framework combines text-to-image synthesis and image inpainting, using curated prompts and real-world segmentation masks to produce diverse and realistic flood scenes. To overcome the lack of human annotations, we employ an unsupervised pseudo-labeling method that generates segmentation masks based on floodwater appearance characteristics. We further introduce a filtering stage based on outlier detection in feature space to improve the realism of the synthetic dataset. Experimental results on five state-of-the-art flood segmentation models show that synthetic data can closely match real data in training performance, and combining both sources improves model robustness by 1–7%. Finally, we investigate the impact of prompt design on the visual fidelity of generated images and provide qualitative and quantitative evidence of distributional similarity between real and synthetic data.

## 1. Introduction

Natural disasters have long affected humanity, with climate change intensifying their frequency and severity. These events cause significant loss of life, property damage, and service disruptions, such as electricity and transportation, while posing serious health risks. The economic and psychological toll is profound [1].

Technological advances such as Pattern Recognition (PR), Deep Learning (DL), Machine Learning (ML), and Artificial Intelligence (AI) provide powerful tools for disaster detection, risk reduction, and response management. As reviewed in [2,3,4], these technologies hold great promise for future disaster response, especially AI and ML in the domain of computer vision (CV), through the use of predictive models that analyze large datasets, identify patterns, forecast potential disasters, and provide early warnings of hazardous events [4,5]. Deep learning (DL) is increasingly used for flood detection and segmentation, overcoming the limitations of traditional mapping [6]. However, DL models require extensive labeled data, which is difficult to obtain in disaster scenarios, requires multiple experts, is time-consuming, and is prone to human annotation.

**Figure 1:**
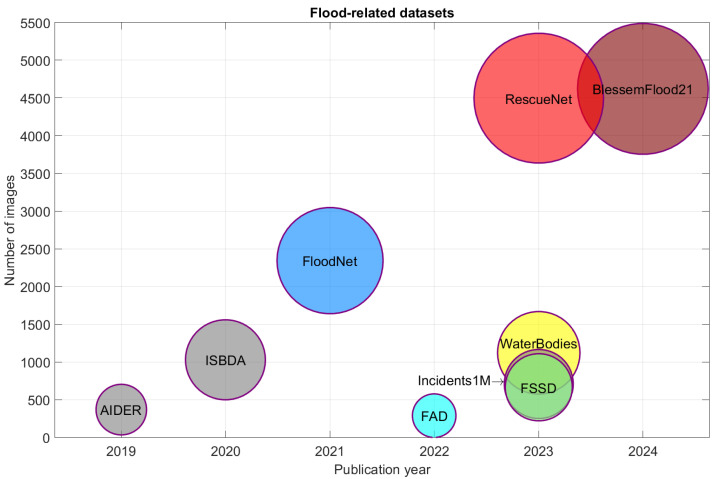
Flood-related aerial image datasets and their sizes (in images). Each bubble has its own color, except for gray bubbles that indicate the absence of explicit flood annotations. Sizes for AIDER and Incidents1M reflect only flood-related images.

To provide a comprehensive overview of the available resources in flood-related visual understanding from aerial imagery, we compiled a selection of ten publicly available datasets, namely AIDER [7], ISBDA [8,9], FloodNet [10], FAD [11], Spacenet-8 [12], FSSD [13], WaterBodies [14], Incidents1M [15], RescueNet [16], and BlessemFlood21 [17] released between 2019 and 2024. These are illustrated in Figure 1, which depicts a bubble chart where each bubble represents a dataset. The horizontal axis indicates the year of publication, while the vertical axis corresponds to the dataset image size. The area of each bubble is scaled proportionally to the dataset size to emphasize the disparity in scale across datasets. Each bubble is also annotated with the dataset name, and a distinct color is used for visual separation. Gray bubbles indicate datasets without flood annotations (AIDER [7], ISBDA [8,9], Incidents1M [15]), and for AIDER [7] and Incidents1M [15] only the subset of flood-related aerial images is considered in the reported sizes.

The increasing trend in dataset size over recent years highlights the growing effort to support deep learning-based flood analysis and emergency response using aerial visual data. However, the creation of large-scale annotated datasets remains a significant challenge. Manual annotation is labor-intensive, often requiring the expert interpretation of complex visual patterns. Furthermore, achieving scene diversity is difficult, as the aerial imagery of floods is typically limited to specific geographical regions and events, which may reduce generalizability across diverse flood scenarios.

To overcome these challenges, one viable solution is the use of synthetic data generated through advanced generative methods, such as diffusion models or generative adversarial networks (GANs). Synthetic imagery enables the creation of diverse flooding scenarios, including rare or extreme cases, thereby improving the model’s generalizability and robustness. Moreover, in the absence of ground truth labels, pseudo-labeling through automated segmentation algorithms offers a promising alternative. These methods can generate approximate annotations at scale, significantly reducing the dependence on expert input and enabling the effective training of deep learning models in a weak or unsupervised manner.

**Figure 2:**
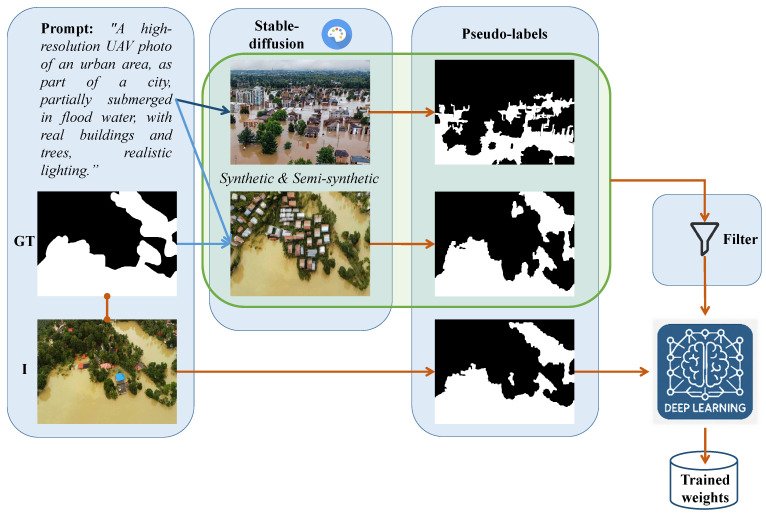
Schematic overview of the proposed framework. Synthetic and semi-synthetic images are generated via Stable Diffusion using prompt engineering, with the latter incorporating original ground truth labels (GT) through image inpainting. Pseudo-labels for original (I), synthetic, and semi-synthetic images are produced via unsupervised segmentation. Outlier filtering is applied, and the combined datasets are used to train deep neural networks for flood segmentation.

In this paper, we solve the problem of flood segmentation in UAV imagery using synthetic generated images of flooding events instead of real ones to answer the following open question: Can pseudo-labels from synthetic UAV data enable real-world flood segmentation? In our work, we propose a framework to create synthetically generated images of flooding events using two algorithms: (i) text-to-image synthesis and (ii) image inpainting based on real segmentation masks. The generated images undergo unsupervised pseudo-labeling for flood segmentation masks, and filtering using feature embeddings and clustering to refine the synthetic dataset. We train different popular Convolutional Neural Network (CNN) and transformer architectures for flood segmentation. Our results show that these models when trained on both synthetic and real images can achieve higher segmentation accuracy. We evaluate their performance on an unseen test set and compare the results to models trained with human-annotated labels and the unsupervised pseudo-labeling approach. A schematic overview of the proposed framework is shown in Figure 2.

The contributions of this work can be summarized as listed below.

- We introduce, to the best of our knowledge, the first scalable pipeline for the unsupervised generation of synthetic aerial flood imagery, utilizing text-to-image diffusion models guided by semantically enriched prompts. To enable segmentation training without the need for manual annotation, we integrate an unsupervised pseudo-labeling approach [18], which automatically produces segmentation masks by exploiting the distinct color characteristics of floodwater and surrounding background elements.
- We demonstrate through extensive experiments with state-of-the-art flood segmentation models that models trained solely on filtered synthetic data achieve a performance close to real-data-trained models, with minor performance drops, and introduce an approach to combine real and synthetic data in order to boost performance.
- We systematically examine how the structure and semantics of text prompts affect the quality and realism of the generated flood imagery, identifying factors that influence scene consistency and visual fidelity.

The remainder of this paper is structured as follows: Section 2 provides a summary of related research. Our proposed unsupervised framework is described in Section 3. Section 4 outlines the experimental framework of this study. Section 5 presents the experimental results along with an in-depth discussion. Finally, Section 6 concludes the paper and outlines directions for future research.

## 2. Related Work

**Flood segmentation in UAV and satellite imagery:** Deep learning techniques, particularly CNNs, are increasingly used for flood segmentation in remote sensing imagery, surpassing traditional methods by enabling the more accurate and efficient delineation of flooded areas and enhancing decision-making processes [6]. CNNs have shown strong capabilities in flood detection from satellite imagery by leveraging temporal variations in synthetic aperture radar (SAR) and multispectral data to differentiate between permanent water bodies and flood-affected areas [19,20]. However, their effectiveness is often constrained by the reliance on pre-disaster imagery for accurate change detection. To address uncertainty in SAR-based water segmentation, Bayesian CNNs have been proposed due to their ability to estimate both the mean and variance of model parameters, providing a probabilistic understanding of predictions [21].

U-Net variants have been widely adopted for water body segmentation and flood extent mapping tasks. For instance, in [22], a modified U-Net architecture was proposed that incorporated geomorphic features and utilized pre-processed Sentinel-1 radar imagery to achieve three-class classification. This model successfully differentiated flood water from permanent water and background. Similarly, in [12,23], it was demonstrated that lightweight U-Net configurations can offer an effective balance between accuracy, computational efficiency, and robustness. The use of transfer learning and targeted data augmentation proved essential in enabling the detection of flooded infrastructure, including roads and buildings. Furthermore, in [14], the performance of various CNN architectures for water body semantic segmentation was evaluated using high-resolution satellite and aerial imagery. The U-Net model with a MobileNet-V3 backbone, along with auxiliary features and data augmentation, achieved superior segmentation accuracy.

Benchmark experiments involving semantic segmentation have validated state-of-the-art deep learning models including XceptionNet and ENet for distinguishing floodwaters from natural water bodies, and detecting inundated roads and buildings in UAV-acquired high-resolution post-disaster imagery [10]. Also, a CNN integrated into the Deep Earth Learning, Tools, and Analysis (DELTA) framework achieved high precision and recall for water segmentation across diverse datasets [24]. In [25], a multiscale attentive decoder network (ADNet) was proposed for automatic flood identification using Sentinel-1 images. When evaluated on the Sen1Floods11 benchmark dataset, ADNet outperformed recent deep learning and threshold-based approaches.

In [26], an enhanced version of the efficient neural network (ENet) architecture was adopted for the semantic segmentation of UAV footage captured during flood events. The approach integrates atrous (dilated) separable convolutions in the encoder, enlarging the receptive field without increasing computational complexity [27], and depth-wise separable convolutions in the decoder, enabling efficient feature extraction with a reduced number of parameters. Atrous convolutions have been further utilized in disaster response scenarios to enhance the efficiency of search and rescue operations during events such as floods, high tides, and tsunamis. A notable example is FASegNet, a recently proposed CNN architecture designed for the semantic segmentation of flood- and tsunami-affected areas [28].

Transformer-based architectures have also demonstrated strong performance in the semantic segmentation of remote sensing imagery. In [29], a novel approach was introduced, employing the Swin Transformer as the backbone to enhance contextual feature representation, coupled with a densely connected feature aggregation module as the decoder. Additionally, the Bitemporal Image Transformer (BiT) model proposed in [19] showed superior performance in change detection tasks by effectively identifying and localizing regions of change between image pairs.

In [30], an interactive semantic segmentation model for multi-source UAV flood images using four prompt types was presented. A prompt encoder maps prompts into a three-channel space to lower labeling costs, while an image encoder, combining Mamba and convolution operations, extracts global features. The model further improves prompt utilization by incorporating a spatial and channel attention module with residual connections. This enables a multiscale fusion and filtering of prompt information and image features across both spatial and channel dimensions.

Several unsupervised flood segmentation methods have been developed using clustering and region-growing techniques. These include object-based K-means with region growing on SAR data [31] and UAV imagery [32], datacube-based flood mapping with probabilistic thresholds [33], tile-based histogram thresholding with contextual filters [34], and graph-based segmentation using Bayesian Markov random fields [35], all demonstrating effectiveness across various imaging sources and scenarios.

In [18], a fully unsupervised segmentation method, UFS-HT-REM, was introduced for fast and accurate flood area detection using UAV-acquired color imagery without requiring pre-disaster reference images. This framework addresses flood segmentation through a parameter-free, unsupervised image analysis pipeline that progressively eliminates non-flood regions using binary masks derived from color and edge information. Specifically, non-flood areas are excluded through mask calculations applied to each channel of the LAB color space, an RGB-based vegetation index, and edge maps from the original image. A probability map of flood presence is then generated using a weighted fusion strategy, followed by a modified hysteresis thresholding process to produce the final segmentation. The method demonstrates both high accuracy and computational efficiency, making it well suited for real-time, on-board processing during UAV operations. This methodology has been used as pseudo-label generator for an unsupervised DL approach [36], and also serves the same purpose as a module in this work.

**Pseudo-Label-Based Methods for Semantic Segmentation:** Pseudo-labels in image segmentation are a common strategy in semi-supervised and unsupervised learning. The idea is to use automatically generated labels, often produced by an initial model, unsupervised method, or a rule-based system to train deep learning models without requiring large amounts of manually annotated data. This approach has been widely explored in general semantic segmentation tasks, such as urban scene understanding, medical imaging, and object detection.

A recent review and analysis of various PL methods and their applications in semi-supervised semantic segmentation (SSSS) underlined that training with limited labeled data by leveraging automatically generated labels can be effective [37]. Limitations of existing pseudo-label generation by leveraging enhanced class activation maps and dual attention mechanisms to produce semantically rich labels, achieving competitive or superior performance, has been addressed in [38]. Furthermore, self-supervised learning and pseudo-label refinement were integrated in a novel weakly supervised semantic segmentation (WSSS) approach, achieving near fully supervised performance by enhancing feature representation and mitigating label noise [39]. In [40], PseudoSeg was introduced, a method that generates structured pseudo-labels for training with unlabeled or weakly labeled data, demonstrating effectiveness in both low-data and high-data regimes.

In the context of flood segmentation, pseudo-labeling is less established. A few recent studies have started exploring automatic label generation for SAR and optical imagery, particularly when annotated flood datasets are limited or unavailable. A semi-supervised learning method for flood segmentation using Sentinel-1 SAR imagery, employing a cyclical training process with an ensemble of U-Net models trained on both high-confidence hand-labeled data and generated pseudo-labels was introduced in [41]. Moreover, an unsupervised deep learning framework for water extraction from multispectral imagery, combining NDWI with a binarization algorithm to generate pseudo-labels for training, was proposed [42]. A novel WSSS framework, TFCSD, has been introduced for efficient urban flood mapping, significantly reducing manual annotation by decoupling the generation of positive and negative samples [43]. The method enhances edge delineation and stability and maintains high performance even without pre-disaster data by incorporating SAM-assisted interactive labeling [44].

In our recent work [36], we proposed a novel unsupervised deep learning approach for flood segmentation in Unmanned Aerial Vehicle (UAV) imagery, which leverages automatically generated pseudo-labels as training and validation masks, thereby eliminating the need for manually annotated ground truth data. Two widely used Convolutional Neural Network (CNN) architectures for semantic segmentation were trained under this framework. The results demonstrated that training with pseudo-labels alone can achieve performance levels comparable to those obtained using conventional ground truth annotations. Finally, in [45] a semi-supervised semantic segmentation algorithm for accurate flood delineation in SAR data was proposed. The method exhibited promising results utilizing a pseudo-label generation strategy and self-supervised teacher–student models.

**Deep generative models and diffusion-based image synthesis:** The performance of modern DL models is fundamentally tied to the availability of large-scale datasets. In many real-world domains, such as disaster monitoring or remote sensing, data acquisition is costly. To address this bottleneck, a promising direction is the generation of synthetic data, which can serve as a scalable and controllable alternative to real-world data collection. Synthetic data can be created through simulation, procedural generation, or by leveraging deep generative models. Among generative approaches, models are typically grouped into four main categories: variational autoencoders (VAEs), generative adversarial networks (GANs), auto-regressive models, and, more recently, diffusion models [46,47].

Diffusion models have emerged as the most widely adopted family of models, in a variety of domains such as image and text-to-image synthesis [48,49,50], image inpainting [47,51], and image-to-image translation [52,53]. These models operate by learning to denoise a sample starting from pure Gaussian noise, gradually transforming it into a realistic image. They exhibit greater training stability compared to GANs and offer fine-grained control over the generation process via conditioning mechanisms, such as textual prompts or image masks, and have proven to generate sharper images compared to VAEs, which often produce blurry outputs due to their reliance on approximate posterior inference [46]. The ability of diffusion-family models to learn strong latent representations that are aligned with textual prompts, and are able to generalize to unseen scenarios (textual prompts) generating high-fidelity images is asserted by the emergence of widely exploited models such as the DALL-E series [54,55], Stable Diffusion [47], and MidJourney, which generate high-quality, customizable images from textual descriptions. Their capacity to synthesize diverse, domain-specific imagery makes diffusion models particularly suitable for augmenting datasets in applications where labeled data is scarce or costly to produce.

## 3. Synthetic Dataset Generation Methodology

Our proposed approach is a framework that enables the construction of a synthetic dataset consisting of UAV images and corresponding segmentation masks for the task of flood segmentation. To achieve this, we employed two distinct generative strategies, subsequently integrating their outputs into a unified dataset. In both strategies, we ensured that the generated images comprehensively represent the range of flood scenarios observed in real-world datasets (see Figure 1). This was achieved by systematically varying key semantic attributes including scene context (urban/peri-urban vs. rural), camera viewpoint (presence or absence of sky), and flood severity (from light inundation to extensive submersion).

**Figure 3:**
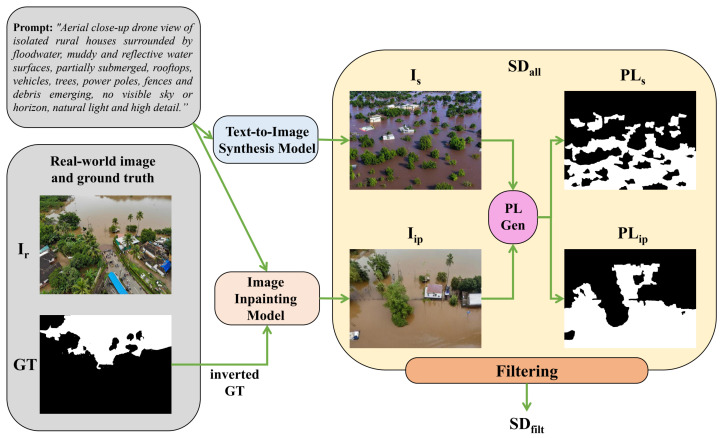
Overview of the synthetic dataset creation pipeline. Synthetic flood images are generated via (i) text-to-image synthesis and (ii) image inpainting guided by real segmentation masks (GT) from real images (I_*r*_). Unsupervised pseudo-labeling (PL Gen) yields segmentation masks PL_*s*_ and PL_*ip*_, for synthetic (I_*s*_) and semi-synthetic (I_*ip*_) images, respectively. The resulting dataset (SD_*all*_) is refined through feature-based clustering to remove outliers, producing the final synthetic dataset (SD_*filt*_) used for training.

The first approach leverages a text-to-image generative model, enabling the synthesis of UAV imagery that encapsulates specific flood-related characteristics, explicitly defined through textual descriptions. The second approach employs an image-to-image translation paradigm, wherein segmentation masks derived from real data serve as structural constraints for synthesizing realistic UAV images via an inpainting method. As a final step in the synthetic dataset creation pipeline, in order to ensure the fidelity and realism of the generated synthetic images, we apply a filtering process, based on outlier detection mechanisms, that aims to discard unrealistic synthetic images. An overview of the synthetic flood dataset generation method is shown in Figure 3.

For both generative strategies, we selected Stable Diffusion [47] over other synthetic data generation methods. Although VAEs and GANs are also common choices for synthetic data generation, Stable Diffusion presents advantages in image quality, training stability, and controllability across diverse domains [48,56]. It enables fine-grained semantic control via prompt conditioning, supports diverse output generation through simple stochastic sampling, and natively handles both text-to-image and image inpainting tasks. These capabilities allowed us to synthesize realistic and varied flood scenarios with high fidelity, which are difficult to achieve with VAEs and GANs due to their limited controllability and domain consistency [46,57]. Thus, Stable Diffusion was a more suitable choice for generating synthetic data to complement real-world flood datasets.

### 3.1. Text-to-Image Synthesis Component

In the first direction, given a text-to-image synthesis models, noted as $G_{s}$, which in our case was the Stable Diffusion [47], and a prompt $P_{i}$ from a predefined set P, the output of the process is a generated synthetic image $I_{s}$:

(1) $$I_{s}=G_{s}\left(P_{i}\right),\;P_{i}\in P.$$

**Figure 4:**
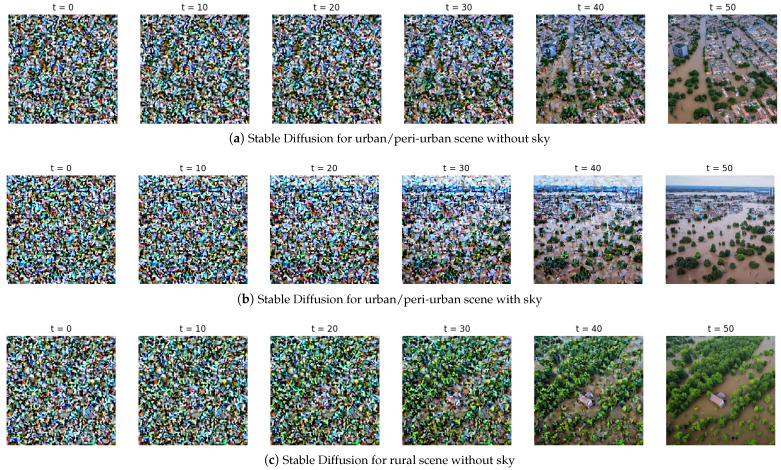

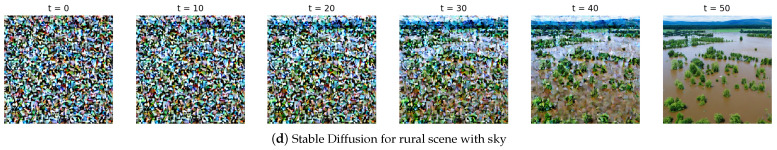
Illustration of the image generation process using Stable Diffusion over 50 denoising steps. The sequence shows the initial random noise and intermediate outputs at every 10th step (t) until the final synthesized image. Diversity in the generated outputs is achieved through carefully constructed text prompts. The model successfully generates realistic flooded environments, including urban/peri-urban scenes (**a**,**b**), and rural landscapes (**c**,**d**). Variations in sky presence which can include sky (**b**,**d**) or not (**a**,**c**), simulate different camera viewpoints and orientations.

Stable Diffusion [47] is a generative model that synthesizes high-quality images by reversing a gradual noising process through a series of denoising steps. Initially, an image is represented as pure noise, and the model iteratively refines it over a fixed number of steps, by progressively removing noise while conditioning on a text prompt or other guidance (e.g., semantic maps or class labels). At each step, the model predicts the noise component and subtracts it from the current image estimate, gradually revealing a coherent image that aligns with the input conditions. We have found that 50 steps are enough to generate a visually realistic image that reflects the intended structure and content, in our case, a flooded environment, generated from an entirely random starting point, as depicted in Figure 4.

The quality of the generated outputs and the ability of the synthetic image dataset to adequately capture the variation inherent in UAV images depicting flooded areas are heavily influenced by the chosen input prompts. To ensure that the dataset encompasses a diverse range of scenarios, we systematically varied the prompts along three key attributes, while controlling flood severity with specific word choices (*partially*, *fully*, *some affected by flooding*, etc.):

- **Rural vs. urban/peri-urban environment**: The images were conditioned to represent either a rural or urban/peri-urban landscape.
- **Sky presence**: The synthesized images either included or excluded visible sky regions.
- **Flooded and non-flooded buildings**: We controlled the number of flooded buildings in the generated images to ensure a diverse range of flooding scenarios.

The following are examples of prompts used for data generation:

- *“Aerial view of a flooded urban area with high-rise buildings and streets underwater."*
- *“Drone footage of a rural landscape with scattered houses, some affected by flooding, others dry.”*
- *“Low-altitude remotely sensed image depicting an urban neighborhood with partially submerged homes and roads.”*
- *“UAV view of a countryside area with a river overflow flooding nearby fields and farmhouses.”*

Since the generated outputs lacked corresponding ground truth segmentation masks, we employed an unsupervised mask generation method to derive pseudo-labels (PL). Specifically, to generate the unsupervised pseudo-labels, we relied on the UFS-HT-REM method, as described in [18]. This method autonomously identifies flooded and non-flooded regions based on color and texture properties in aerial imagery. A hysteresis threshold T(·) is applied on a probabilistic map PM(·) derived from color, intensity, and reflectance information CIR(·):

(2) $$PL_{s}=T\left(PM\left(CIR\left(I_{s}\right)\right)\right).$$

where $PL_{s}$ is the resulting pseudo-label binary mask of the synthetic image $I_{s}$, with each pixel value defined as

(3) $$PL_{s}\left(x,y\right)=\left\{\begin{array}{cc} 1, & \mathrm{if}\;(x,y)\;\mathrm{belongs}\;\mathrm{to}\;a\;\mathrm{flooded}\;\mathrm{region}\\ 0, & \mathrm{otherwise}. \end{array}\right.$$

This process ensures robust segmentation by leveraging statistical color distributions and intensity variations to separate flood water from background, even in visually complex images. The final output of the process is a synthetic UAV image $I_{s}$ and its corresponding binary pseudo-label mask $PL_{s}$.

### 3.2. Image Inpainting from Segmentation Masks Component

An alternative approach to generating synthetic UAV images was image inpainting using real segmentation masks. This method involved using segmentation masks from a real dataset and applying an inpainting algorithm to synthesize realistic UAV imagery corresponding to the given segmentation structure. Using real flood segmentation masks as priors provides a more grounded structural context, ensuring that the generated images reflect plausible flood extents and spatial layouts as observed in real-world scenarios. This leads to synthetic data that are more representative and structurally aligned with actual flood patterns, thereby improving its utility for training segmentation models.

Overall, the process can be formulated as follows. Given a flood-related image, $I_{r}$, selected from a real-world UAV flood segmentation dataset, and its corresponding ground truth segmentation mask GT, employ an image inpainting model $G_{ip}$ to generate a semi-synthetic UAV image $I_{ip}$ with the real flood area untouched, thus inpainting the labeled background, conditioned on a textual context prompt, $P_{j}$:

(4) $$I_{ip}=G_{ip}\left(GT,P_{j}\right).$$

Following the previous approach, we again adopted Stable Diffusion [47] in our workflow as an image inpainting model in a text-guided image-to-image translation framework. The prompts were selected based on the image category, ensuring consistency with the expected scene semantics. Prompt context was similar to the previous process, to ensure a thematic consistency between both synthetic image generation schemes.

Since the generated image $I_{ip}$ does not always exhibit a perfect one-to-one correspondence with the input ground truth GT, we apply the unsupervised pseudo-labeling method once again to derive a refined segmentation mask $PL_{ip}$ for the newly synthesized image:

(5) $$PL_{ip}=T\left(PM\left(CIR\left(I_{ip}\right)\right)\right).$$

We observed that this process better ensures that the final segmentation pseudo-labels $PL_{ip}$ more accurately reflect the flooded and non-flooded regions present in the semi-synthetic, inpainted images.

### 3.3. Post-Generation Filtering for Enhanced Dataset Fidelity

Following the generation of two synthetic image sets

(6) $$SD_{s}=\left\{\left(I_{s},PL_{s}\right)\;|\;I_{s}=G_{s}\left(P_{i}\right),\;\;PL_{s}=T\left(PM\left(CIR\left(I_{s}\right)\right)\right)\right\},$$

and

(7) $$SD_{ip}=\left\{\left(I_{ip},PL_{j}\right)\;|\;I_{ip}=G_{ip}\left(GT,P_{j}\right),\;\;PL_{ip}=T\left(PM\left(CIR\left(I_{ip}\right)\right)\right)\right\},$$

we merged both sets into a unified synthetic dataset:

(8) $$SD_{all}=SD_{s}\cup SD_{ip}.$$

To improve the unified synthetic dataset fidelity, we applied the FINCH [58,59] clustering algorithm to $SD_{all}$, retaining only images from the first partition and discarding those that formed singleton clusters. FINCH is an ideal choice since it is a parameter-free hierarchical clustering algorithm that constructs clusters in a cluster-number agnostic way using a simple yet effective strategy based on first-neighbor relations. Specifically, each data point is initially linked to its nearest neighbor, and transitive links are formed such that if point A links to B and B links to C, all three belong to the same cluster. This process yields a partitioning of the data without requiring the number of clusters or other hyperparameters to be specified. The procedure is applied recursively to the resulting centroids, producing a hierarchy of clusterings that capture increasingly abstract groupings.

This choice ensures that filtering is applied early in the generation pipeline, where redundancy and noise are more prevalent, thereby preventing low-quality or outlier samples from propagating into later stages. Next, we encoded each synthetic image into a feature vector of size 1×1024 using a pre-trained ResNet50 model.

To further refine the dataset, we applied FINCH clustering to a real-world dataset and extracted cluster centers. We then computed pairwise Euclidean distances between the real cluster centers and the synthetic image feature vectors. Synthetic images were assigned to the closest cluster, and those failing to align with any real-world cluster were discarded:

(9) $$d\left(I_{syn},C_{real}\right)=\min_{c}||f\left(I_{syn}\right){-c∥}_{2},\;\;c\in C_{real}$$

where $f(I_{syn})$ represents the feature embedding of a synthetic image and $C_{real}$ is the set of real-world cluster centers.

After applying the filtering process, where images failing to align with real-world cluster structures are discarded, the final filtered synthetic dataset $SD_{filt}$ is defined as

(10) $$SD_{filt}=\left\{\left(I,PL\right)\in SD_{all}|d\left(f\left(I\right),C_{real}\right)\leq \tau \right\},$$

where *I* is a synthetic or semi-synthetic image, and PL is its corresponding pseudo-label segmentation mask, f(I) represents the feature embedding of the image *I*, extracted using a pre-trained ResNet50, $C_{real}$ is the set of cluster centers obtained from the real-world dataset using the FINCH algorithm, d(·,·) denotes the Euclidean distance between the synthetic image embedding and the closest real-world cluster center, and τ is a distance threshold that determines the inclusion of synthetic images based on their proximity to the real-world data. The resulting set $SD_{filt}$, has a size of 557 images (279 from SD_*s*_, 278 from SD_*ip*_) and their corresponding pseudo-label masks, whereas pre-filtering size was 580 images (290 from SD_*s*_, 290 from SD_*ip*_) and their corresponding pseudo-label masks. Synthetic image sets are publicly available (https://sites.google.com/site/costaspanagiotakis/research/flood-detection, accessed on 3 June 2025).

**Figure 5:**
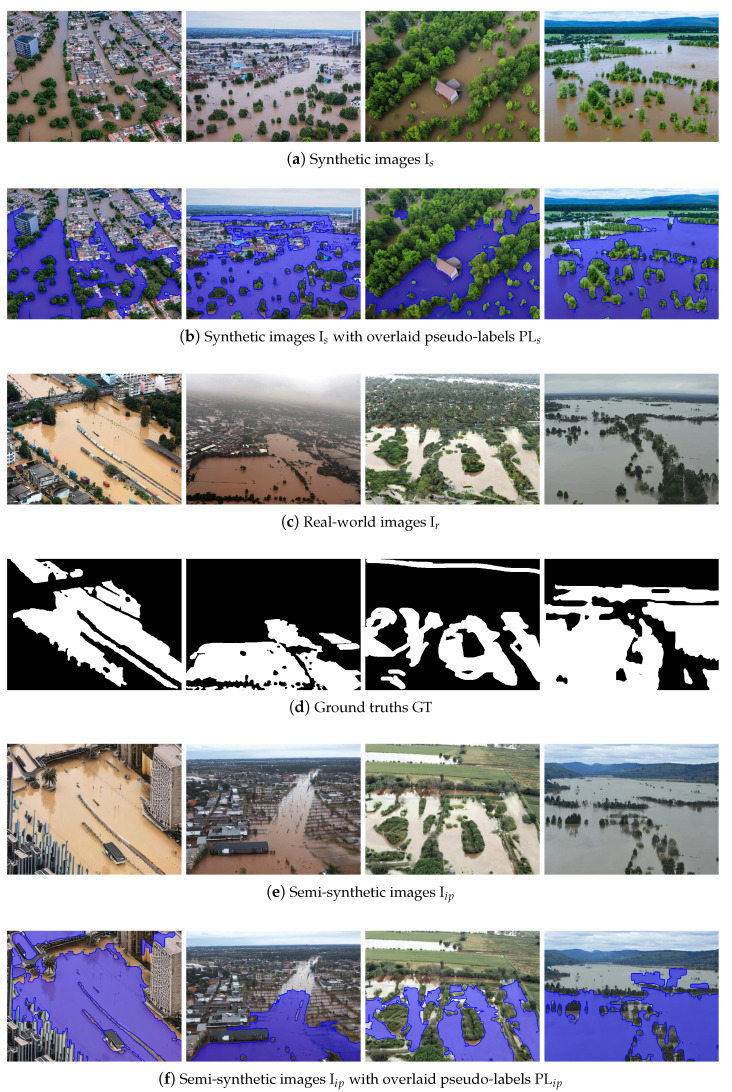
Synthetic images $I_{s}$ (**a**) from Figure 4 representing flooded urban/peri-urban environments with sky absence and presence, and rural scenes also without and with sky. Real-world UAV captured flood-related images with the same scene diversity (**c**), along with their respective ground truths (**d**), which are used, as described in Section 3.2, to generate semi-synthetic images $I_{ip}$ (**e**) via inpainting. Respective pseudo-labels, PL_*s*_ and PL_*ip*_, are overlaid in blue for the synthetic I_*s*_ (**b**) and semi-synthetic images I_*ip*_ (**f**).

In Figure 5, representative examples of synthetic (a) and semi-synthetic (e) images, and their corresponding pseudo-label segmentation masks overlaid in blue, (b) and (f), are shown. For the inpainting process of the semi-synthetic images, ground truths (d) were used, derived from real-world images (c). The synthetic images exhibit a high degree of diversity in terms of environmental conditions, flood severity, and background complexity, as well as variation in camera angles and perspectives. This diversity is essential to enhance the model’s robustness and generalizability across a wide range of flooding scenarios. Moreover, the synthetic data were generated with particular attention to visual realism and structural similarity to real-world flood imagery, enabling the more effective transfer of knowledge when such images are used in training. As previously mentioned, when inpainting is applied, the original ground truth annotations become invalid, as the generative process may not only modify the background but also extend or alter the spatial extent of the flooded regions, thereby introducing discrepancies between the modified image and its initial label.

**Figure 6:**
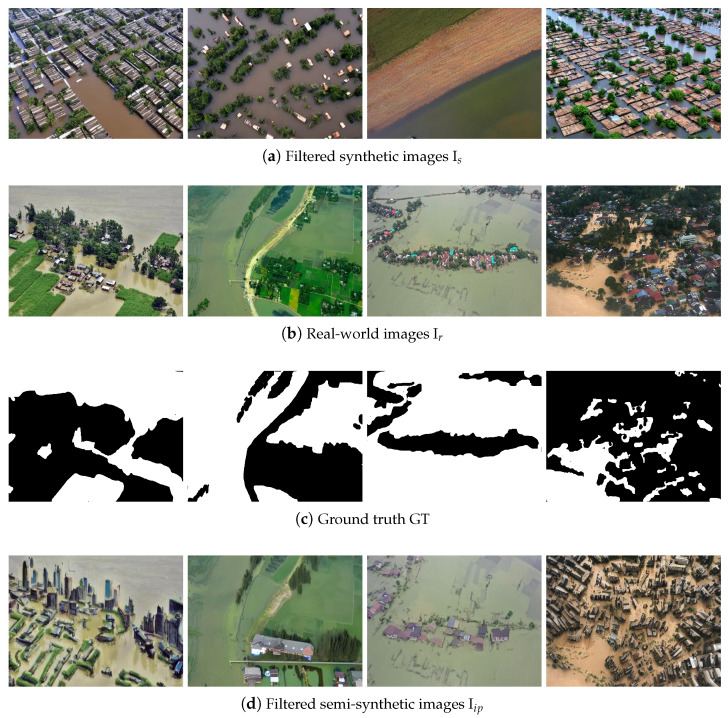
Representative examples of synthetic (**a**) and semi-synthetic images (**d**) which were filtered with outlier threshold k=1.5. Semi-synthetic images $I_{ip}$ were generated as described in Section 3.2, with the ground truth GT (**c**) used for in-painting derived from the real-world UAV captured flood-related images $I_{r}$ (**b**).

Figure 6 presents examples of synthetic (a) and semi-synthetic (d) images which did not pass the filtering process. Also, the real-world images (b) and their respective ground truths (c), from which the semi-synthetic versions were generated, are also provided for reference. The discarded synthetic samples exhibit significant deviations from realistic flood scenes, including structural inconsistencies, the unnatural repetition of patterns, excessive blurriness, or visual characteristics resembling stylized artwork (e.g., oil paintings). These artifacts resulted in the images being classified as unrealistic according to our established outlier scoring scheme, thereby justifying their removal from the training set.

**Outlier threshold, τ:** we employ a z-score-based filtering mechanism grounded in the distribution of distances between synthetic features and real cluster centers. Specifically, for each synthetic feature vector, we computed its minimum Euclidean distance to the closest cluster center derived from FINCH clustering on real data. Let μ and σ denote the mean and standard deviation of these minimum distances, respectively. A synthetic sample *i* was retained if its distance $d_{i}$ satisfied:

(11) $$d_{i}\leq \mu +k\cdot \sigma ,$$

where *k* is a hyperparameter controlling the strictness of the filtering (we set k=1.5 in our experiments). This statistical approach adapts the filtering threshold to the inherent distribution of the distances, avoiding arbitrary cutoffs and promoting a more robust selection of semantically similar synthetic samples.

## 4. Experimental Setup

To assess the generalization capability of a segmentation model trained on synthetic flood imagery, we conducted a series of controlled experiments using state-of-the-art deep learning architectures. Specifically, we selected three top-performing segmentation models which were trained under six different data configurations: (a) exclusively on the real-world dataset with the actual ground truth $D_{r}=\left(I_{r},GT\right)$, (b) on the real-world dataset with pseudo-labels assuming no ground truth is available $D_{pl}=\left(I_{r},PL_{r}\right)$, (c) on the synthetic dataset $SD_{s}$ (see Equation (6)) with corresponding pseudo-labels ($PL_{s}$), (d) on the semi-synthetic dataset $SD_{ip}$ (see Equation (7)) with corresponding pseudo-labels ($PL_{ip}$), (e) on the combination of real-world and both synthetic and semi-synthetic datasets $D_{r}\cup SD_{all}$ ($SD_{all}$ as defined in Equation (8)) with the actual ground truth and corresponding pseudo-labels ($PL_{all}$), and (f) on the combined real-world and filtered synthetic and semi-synthetic datasets $D_{r}\cup SD_{filt}$ ($SD_{filt}$ as defined in Equation (10)) with the actual ground truth and corresponding pseudo-labels ($PL_{filt})$. The evaluation was performed on an independent real-world dataset with respective expert annotations, which was not used during training, to measure the effectiveness of the synthetic data in bridging the domain gap.

### 4.1. Datasets and Methods

**Datasets:** For the real-world dataset baselines, we employed two publicly available datasets that depict flood-affected regions, each accompanied by ground truth segmentation masks delineating flooded areas. These datasets consist of aerial imagery captured by UAVs and helicopters, encompassing a diverse range of environmental contexts, including urban, peri-urban, and rural landscapes. The images exhibit significant variability in scene composition, featuring elements such as vegetation, rivers, buildings, roads, mountainous terrain, and the sky. Additionally, they were acquired from multiple altitudes and viewing angles, ensuring a comprehensive representation of flood scenarios. Notably, both datasets maintain a similar balance between flood and background pixels, which mitigates potential class imbalance issues during model training.

As a baseline training set $D_{r}$, we opted to use the well-known *Flood Area Dataset* (FAD) [11]. This dataset comprises 290 RGB images, accompanied with manually annotated segmentation masks. The dataset exhibits variability in image resolution and dimensions. Contributing to the dataset’s heterogeneity is its diverse range of environmental contexts, including 203 urban and peri-urban scenes and 87 rural scenes. In terms of visual composition, 108 images contain full or partial views of the sky, while 182 images lack any visible sky. This diversity supports robust model training across varying landscape types and viewing conditions.

**Figure 7:**
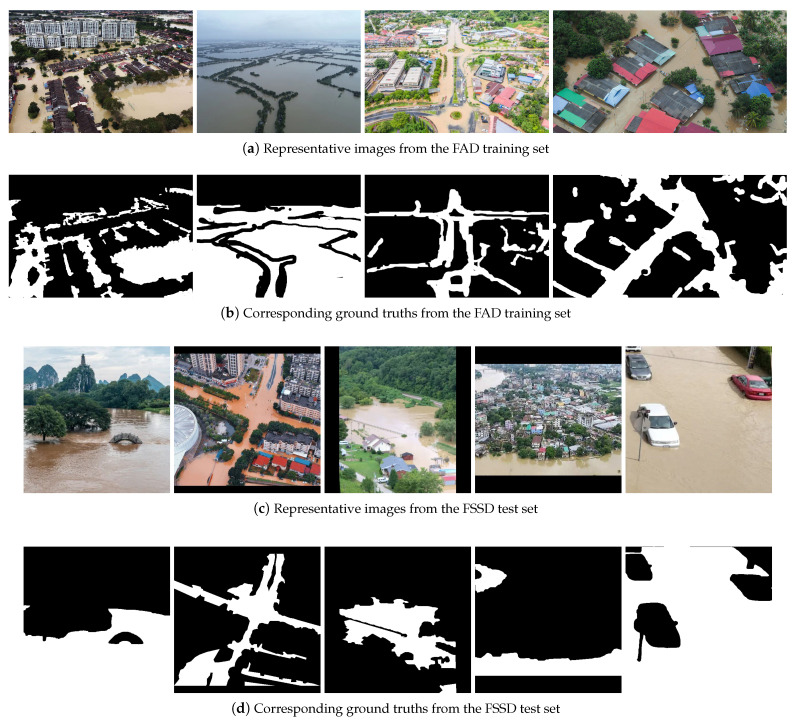
Sample images from the training dataset FAD [11] (**a**) and the corresponding ground truths (**b**), which were used in Section 3.2 to generate semi-synthetic images, and from the test dataset FSSD [13] (**c**) together with their respective ground truths (**d**).

To assess the generalization capability of the segmentation models, we utilized only for inference the *Flood Semantic Segmentation Dataset* (FSSD) [13] as an independent test set, which consists of 663 RGB images and corresponding ground truth segmentation masks. Similarly to the FAD, this dataset comprises images obtained from UAVs portraying diverse flooded scenes captured from various camera perspectives. The image sizes and resolutions also vary, but were all resized and, if necessary, zero-padded, to 512 × 512 by the dataset creator. All 663 images were used as our test set. Representative samples from both datasets, along with the corresponding ground truth masks, are illustrated in Figure 7.

**Flood segmentation methods:** We trained three well-established Convolutional Neural Networks (CNNs) for semantic segmentation tasks, and two transformer-based models.

We started with the following CNN architectures: DeepLabV3, FCN-ResNet50, and U-Net. DeepLabV3 [60] incorporates atrous (dilated) convolution and Atrous Spatial Pyramid Pooling (ASPP) to capture multi-scale contextual information without reducing spatial resolution. Built on a ResNet backbone, it is effective in segmenting complex scenes and fine structures. FCN-ResNet50 follows the Fully Convolutional Network design [61] enhanced with a ResNet-50 backbone. This architecture performs dense prediction by replacing fully connected layers with convolutions, while residual and skip connections help preserve spatial detail. U-Net, introduced in [62], features a symmetric encoder–decoder structure with skip connections between corresponding layers. This design enables precise localization and efficient learning from limited data, making it especially suitable for segmentation tasks.

Furthermore, we included two Transformer-based models in our evaluation: SegFormer-B0 and Swin-Tiny with UPerNet (Swin-T). SegFormer-B0 [63] combines a lightweight MixVision Transformer encoder with a UPerNet-style decoder. The encoder avoids positional embeddings for improved generalization and employs a hierarchical design that efficiently captures multi-scale context. The B0 variant offers a strong performance–efficiency trade-off, making it suitable for training from scratch with limited data while maintaining good segmentation quality. Swin-T leverages the Swin Transformer’s hierarchical architecture with shifted window attention, enabling the effective modeling of both local and global dependencies [64]. Coupled with the UPerNet decoder, which aggregates multi-scale features, this model delivers a strong performance in dense prediction tasks and excels at delineating sharp object boundaries, but at a greater computational cost.

### 4.2. Training Protocol

**Implementation Details:** From the range of diffusion models, we selected Stable Diffusion due to its superior ability to generate photorealistic aerial and environmental flood scenes that closely resemble UAV imagery, including urban and rural landscapes with varied perspectives (e.g., top–down, oblique). Unlike models such as Midjourney or DALL·E, Stable Diffusion offers fine-grained prompt control, open source accessibility, fine-tuning capabilities, native support for image inpainting enabling targeted scene manipulation and seamless integration into PyTorch-based pipelines.

In this study, we employed Stable Diffusion v1.5 [47], chosen for its balance between visual fidelity, controllability, and computational efficiency. Trained on the extensive and diverse LAION-5B dataset [65], this version is particularly effective at generating realistic and coherent environments, accurately capturing natural elements such as water, rain, clouds, and terrain, making it well suited for synthesizing UAV-like flood imagery. Compared to earlier versions, it offers improved stability, reduced artifacts, and greater prompt interpretability, supporting prompts up to 77 tokens, which enabled us to steer the generation process toward specific flood scenarios (e.g., “urban flooding” or “flooded rural roads with debris”). These characteristics ensure high-quality synthetic data with realistic textures and geometries, which is critical for effective training of segmentation models. Additionally, the relatively compact architecture of the model (860 M UNet, 123 M text encoder) allows for efficient deployment on consumer-grade GPUs.

All models were implemented in PyTorch (v. 2.6.0, CUDA 12.6) and trained from scratch for 100 epochs with a random generated batch of eight images, using an initial learning rate of $1\times 10^{-3}$. Each utilized dataset was randomly partitioned into 90% for training and 10% for validation. The Dice loss function was employed as the objective to address class imbalance, and model performance was evaluated using the accuracy on the validation set.

For the CNN models, a dynamic learning rate adjustment strategy was employed, whereby the learning rate was reduced by a factor of 0.5, if no improvement in validation performance was observed over five consecutive epochs. Optimization was conducted using the Adam optimizer with a weight decay of $1\times 10^{-4}$ to prevent overfitting by penalizing large weights and encouraging model generalization. Adam was selected due to its adaptive learning rate strategy, which computes individual learning rates for each parameter based on the estimates of first and second moments of the gradients. This facilitates efficient convergence and robustness in training deep neural networks, particularly in high-dimensional and non-convex optimization landscapes. The model weights were initialized using the Kaiming normal initialization, which is specifically designed for layers with ReLU activations. This initialization strategy maintains the variance of activations across layers, thereby promoting stable gradient flow during training and preventing issues such as vanishing or exploding gradients.

For the transformer-based models, we followed the suggested training configurations of the original papers [63,64], and utilized AdamW as the optimizer with a weight decay of $1\times 10^{-2}$, and a cosine learning rate scheduler with warm-up. AdamW is a variant of Adam that decouples weight decay from the gradient update. This modification improves generalization and is particularly effective for training transformer architectures. Like Adam, it adapts learning rates based on the first and second moments of the gradients, enabling stable and efficient optimization in complex, high-dimensional settings. The cosine learning rate scheduler with warm-up gradually increases the learning rate during an initial warm-up phase, followed by a cosine decay, which helps prevent early training instability and encourages smoother convergence. The model weights were initialized using the standard procedures provided by the respective implementations, without any pre-training.

Notably, improved model convergence and higher validation accuracy were observed when training was performed on a combined dataset comprising real-world, synthetic, and semi-synthetic images (see also Section 5). In this setting, each training batch was composed of a fixed ratio of samples—specifically, two randomly selected images from the real-world dataset and three images each from the synthetic and semi-synthetic subsets. This sampling strategy enabled the model to benefit from the diversity and volume of synthetic data while maintaining grounding in real-world examples, effectively enhancing generalization and training stability.

Training was performed on a system equipped with an Intel i7 CPU (2.3 GHz), 40 GB of RAM, and two NVIDIA Quadro RTX 4000 GPUs. The total training times ranged from less than an hour to approximately four hours, depending on the deep learning architecture and the size of the training dataset.

**Dataset pre-processing:** In the case of model training in real-word data, as previously stated, we utilized the FAD dataset. In the absence of ground truth, corresponding pseudo-label segmentation masks were automatically generated using an adapted UFS-HT-REM [18] pipeline. All images were resized to 416×320 pixels to speed up training, and normalized to zero mean and unit variance. These processes were also applied on the synthetic dataset, as well as on the FFSD dataset that is used for model performance evaluation.

**Data augmentation:** To mitigate the limited training data, we applied on-the-fly augmentation with a per-transformation probability of 0.5. Augmentations included horizontal flipping, random rotations within [−15,15] degrees, and additive uniform noise in the range [−0.2,0.2]. Over 100 epochs, this strategy effectively expanded training variability.

## 5. Experimental Results

In this section, we present an evaluation of the proposed synthetic dataset generation method in the UAV flood segmentation task. First, we assess the effectiveness of training segmentation models using only synthetic data, comparing their performance against the same models trained on real-world datasets. In the second part of this section, we analyze the role of prompt structure and semantic context in the quality and utility of the generated synthetic images. Additionally, we evaluate the similarity between the synthetic and real-image feature distributions by providing clustering statistics.

### 5.1. Impact of Synthetic Data on Model Performance

We evaluated the impact of the proposed synthetic dataset creation framework on the performance of three widely used convolution-based semantic segmentation architectures: DeepLabV3 [60], FCN-ResNet50 [61], and U-Net [62], and on two top-performing transformer-based architectures, namely SegFormer-B0 [63] and Swin-Tiny + UPerNet (Swin-T) [64]. For this, each model was trained exclusively on both intermediate synthetic datasets (text-to-image, $SD_{s}$, and image inpainting, $SD_{ip}$), on the combined real-world and synthetic dataset, before ($D_{r}\cup SD_{all}$) and after the filtering process ($D_{r}\cup SD_{filt}$), and was compared against its counterpart trained on the real-world dataset with the actual ground truth $D_{r}$, and with pseudo-label segmentation masks $D_{pl}$ instead of the ground truth. As reported in Table 1, Table 2 and Table 3, across all convolutional architectures, we observed that models trained solely on synthetic data achieved segmentation performance remarkably close to those trained on real-world data, and in case of the U-Net even better. On average, the observed performance was above 70% in F_1_-score, with a drop in the range of 2–5% compared to training with real-world data, and a raise of 1.07% in case of the U-Net, highlighting the high fidelity and generalizability of the generated synthetic samples.

A similar performance impact is observed for the transformer-based architectures, as shown in Table 4 and Table 5, where on average performance was also above 71% in F_1_-score. In these cases, a model trained solely on synthetic data, resulted in an average performance difference of 4% compared to a version trained on real-world data (rows 3 and 5–6 in Table 4 and Table 5). Interestingly, while the general performance trend between real and synthetic datasets holds consistently across both convolutional and transformer-based models, the absolute segmentation performance of the transformer-based architectures was found to be comparable to or slightly lower than that of the convolutional models. This can be partly attributed to the fact that all models were trained under identical learning configurations, which might have not allowed for the transformer-based methods to reach their full potential, given the fact that recent findings suggest that transformer-based architectures typically require longer training schedules to fully converge, due to their higher capacity and weaker inductive biases. Moreover, transformers tend to benefit more from large, diverse datasets, because of their global attention mechanisms, whereas convolutional models can generalize better under limited data due to their strong local spatial priors.

**Table 1:** DeepLabV3 performance on the test dataset (FSSD) based on training with the real dataset and ground truths ($D_{r}$), the real dataset and pseudo-labels ($D_{pl}$), the text-to-image synthetic dataset ($SD_{s}$), the semi-synthetic image inpainting dataset ($SD_{ip}$), and their union, unfiltered ($SD_{all}$) and filtered ($SD_{filt}$), when it is combined with the real-world dataset $D_{r}$. The best-performing values for each metric are highlighted in bold.

Training	Test Metrics				
**Dataset**	**Acc (%)**	**IoU (%)**	**Pr (%)**	**Rec (%)**	**F_1_ (%)**
D_*r*_	79.48	60.03	**67.39**	84.62	75.03
D_*pl*_	78.03	58.75	65.04	85.85	74.01
SD_*s*_	76.48	55.02	64.47	78.97	70.99
SD_*ip*_	74.54	54.70	60.85	84.42	70.72
D_*r*_ ∪ SD_*all*_	79.21	60.94	65.89	89.02	75.73
D_*r*_ ∪ SD_*filt*_	**79.55**	**61.34**	66.34	**89.06**	**76.04**

Among the evaluated architectures, we observed that DeepLabV3 exhibited the highest performance drop when trained solely on synthetic data, while U-Net consistently achieved a slight increase in performance—approximately 1% across all evaluation metrics—compared to its training on real-world data. This divergence in behavior can be attributed to architectural differences: DeepLabV3, with its atrous spatial pyramid pooling and deeper encoder, may rely more heavily on fine-grained real-world features and textures that the synthetic data only partially captures. In contrast, U-Net’s symmetric encoder–decoder structure with skip connections may better exploit the spatial coherence and regularity present in the synthetic masks and images, thus benefiting from the structured nature of the generated dataset.

**Table 2:** FCN-ResNet50 performance on the test dataset (FSSD) based on training with the real dataset and ground truths ($D_{r}$), the real dataset and pseudo-labels ($D_{pl}$), the text-to-image synthetic dataset ($SD_{s}$), the semi-synthetic image inpainting dataset ($SD_{ip}$), and their union, unfiltered ($SD_{all}$) and filtered ($SD_{filt}$), when it is combined with the real-world dataset $D_{r}$. The best-performing values for each metric are highlighted in bold.

Training	Test Metrics				
**Dataset**	**Acc (%)**	**IoU (%)**	**Pr (%)**	**Rec (%)**	**F_1_ (%)**
D_*r*_	83.60	66.90	71.65	90.98	80.16
D_*pl*_	83.64	67.67	70.72	94.00	80.72
SD_*s*_	81.35	64.80	67.48	**94.22**	78.64
SD_*ip*_	81.28	64.44	67.65	93.14	78.38
D_*r*_ ∪ SD_*all*_	83.74	67.82	70.84	94.09	80.83
D_*r*_ ∪ SD_*filt*_	**84.22**	**68.12**	**72.08**	92.54	**81.04**

**Table 3:** U-Net performance on the test dataset (FSSD) based on training with the real dataset and ground truths ($D_{r}$), the real dataset and pseudo-labels ($D_{pl}$), the text-to-image synthetic dataset ($SD_{s}$), the semi-synthetic image inpainting dataset ($SD_{ip}$), and their union, unfiltered ($SD_{all}$) and filtered ($SD_{filt}$), when it is combined with the real-world dataset $D_{r}$. The best-performing values for each metric are highlighted in bold.

Training	Test Metrics				
**Dataset**	**Acc (%)**	**IoU (%)**	**Pr (%)**	**Rec (%)**	**F_1_ (%)**
D_*r*_	81.03	62.62	68.95	87.21	77.01
D_*pl*_	80.33	63.04	66.65	92.07	77.33
SD_*s*_	81.45	64.04	68.56	90.68	78.08
SD_*ip*_	81.11	64.04	67.63	92.34	78.08
D_*r*_ ∪ SD_*all*_	81.91	65.52	68.21	**94.32**	79.17
D_*r*_ ∪ SD_*filt*_	**86.97**	**72.00**	**76.85**	91.94	**83.72**

**Table 4:** SegFormer-B0 performance on the test dataset (FSSD) based on training with the real dataset and ground truths ($D_{r}$), the real dataset and pseudo-labels ($D_{pl}$), the text-to-image synthetic dataset ($SD_{s}$), the semi-synthetic image inpainting dataset ($SD_{ip}$), and their union, unfiltered ($SD_{all}$), and filtered ($SD_{filt}$), when it is combined with the real-world dataset $D_{r}$. The best-performing values for each metric are highlighted in bold.

Training	Test Metrics				
**Dataset**	**Acc (%)**	**IoU (%)**	**Pr (%)**	**Rec (%)**	**F_1_ (%)**
D_*r*_	78.40	61.47	63.70	94.61	76.14
D_*pl*_	79.43	62.43	65.10	93.84	76.87
SD_*s*_	79.03	61.12	65.30	90.52	75.87
SD_*ip*_	74.34	57.63	59.17	**95.68**	73.12
D_*r*_ ∪ SD_*all*_	80.13	63.61	65.66	95.33	77.76
D_*r*_ ∪ SD_*filt*_	**81.22**	**64.96**	**66.99**	95.48	**78.74**

A particularly noteworthy outcome of our experimental analysis is the consistent performance improvement observed across all examined segmentation models, when trained on the combined dataset comprising both real-world data (images and manually annotated masks) and synthetic data (generated images and pseudo-label masks). These gains range between 1% and 6% in the F_1_-score for the convolutional architectures and between 3% and 7% for the transformer-based architectures. This observed behavior underscores the complementary nature of the synthetic dataset. The inclusion of synthetically generated samples, which capture diverse flood-affected scenarios and augment underrepresented visual patterns, likely aids in regularizing the models and enhancing their generalization capabilities. Additionally, the pseudo-label masks, though automatically generated, provide reasonably accurate supervision signals that help the models learn more robust decision boundaries. This result highlights the value of synthetic data as a scalable and effective means to enrich limited annotated datasets in domain-specific applications such as disaster response.

**Table 5:** Swin-T performance on the test dataset (FSSD) based on training with the real dataset and ground truths ($D_{r}$), the real dataset and pseudo-labels ($D_{pl}$), the text-to-image synthetic dataset ($SD_{s}$), the semi-synthetic image inpainting dataset ($SD_{ip}$), and their union, unfiltered ($SD_{all}$) and filtered ($SD_{filt}$), when it is combined with the real-world dataset $D_{r}$. The best-performing values for each metric are highlighted in bold.

Training	Test Metrics				
**Dataset**	**Acc (%)**	**IoU (%)**	**Pr (%)**	**Rec (%)**	**F_1_ (%)**
D_*r*_	81.85	64.93	68.69	**92.22**	78.74
D_*pl*_	81.24	61.66	70.72	82.80	76.28
SD_*s*_	78.67	56.94	68.27	77.43	72.57
SD_*ip*_	77.11	55.98	65.16	79.90	71.78
D_*r*_ ∪ SD_*all*_	83.53	66.39	72.11	89.32	79.80
D_*r*_ ∪ SD_*filt*_	**84.88**	**68.69**	**73.66**	91.06	**81.44**

The introduction of our filtering strategy following the initial creation of the synthetic dataset (row 5 vs. row 6 in Table 1, Table 2 and Table 3) further enhances model performance across all segmentation architectures. Specifically, we observe improvements ranging from 1% to 4% in key evaluation metrics when the filtered synthetic dataset is used instead of the unfiltered one. Among the evaluated models, U-Net consistently exhibits the highest performance gain, suggesting that its architecture may be particularly sensitive to noisy or low-quality training examples. By eliminating synthetic samples with low semantic alignment to real data distributions, the filtering stage effectively increases the signal-to-noise ratio in the training set, leading to better convergence and generalization. This demonstrates the importance of curating synthetic data not only in terms of diversity, but also in maintaining fidelity to real-world distributions.

**Figure 8:**
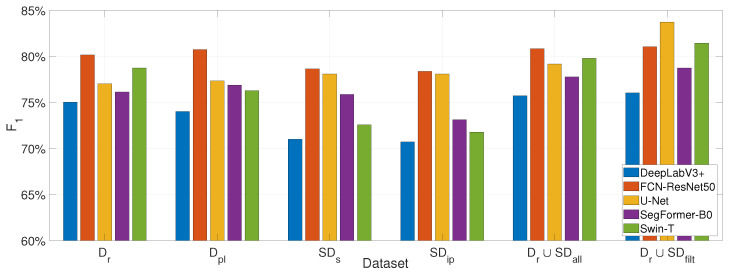
The F_1_-score of DeepLabV3, FCN-ResNet50, U-Net, SegFormer-B0, and Swin-T on the test dataset (FSSD) according to training with the real dataset with ground truth ($D_{r}$) and pseudo-label ($D_{pl}$) masks, text-to-image synthetic dataset ($SD_{s}$), the semi-synthetic image inpainting dataset ($SD_{ip}$), and their union, unfiltered ($D_{r}\cup SD_{all}$) and filtered ($D_{r}\cup SD_{filt}$).

Among the evaluated convolutional architectures, as observed in Figure 8, DeepLabV3 yielded the lowest performance when trained solely on synthetic data, though it was able to marginally surpass training on real-world data when a combined real and synthetic dataset was used. FCN-ResNet50 consistently achieved the highest performance in most scenarios, yet its performance declined slightly under synthetic-only training conditions. U-Net, in contrast, demonstrated notably stronger performance with synthetic data and significantly outperformed the other deep learning models when trained with a filtered combination of real and synthetic data. This can be attributed to U-Net’s encoder–decoder structure with skip connections, which excels at capturing both fine-grained local details and global spatial context. Such architectural characteristics are particularly well-suited for segmentation tasks involving structured patterns. Consequently, the U-Net architecture excels in our synthetic flood imagery that features regular shapes and consistent textural cues, as generated via Stable Diffusion. U-Net’s inductive biases align well with the spatial regularity present in the synthetic data, enhancing its ability to generalize effectively in this context. Statistically, for this model family, comparing the F_1_ scores, we have obtained 58.22% over all images better inferences with U-Net, 29.26% with FCN-ResNet50, and only 12.67% with DeepLabV3 indicating it is the worst performing model when trained with combined real and synthetic data ($D_{r}\cup SD_{filt}$). Note that inferences can excel in more than one model.

**Figure 9:**
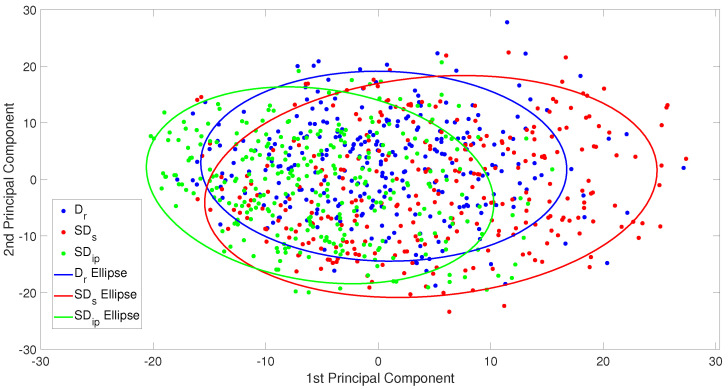
Two-dimensional PCA visualization with blue, red and green colors of ResNet50 feature embeddings extracted from real (D_*r*_) and synthetic (SD_*s*_, SD_*ip*_) datasets, respectively, with the best fit ellipses of each dataset calculated by 2D normal distribution fitting. Each point represents an image, colored by dataset. The substantial overlap suggests strong visual alignment between real and synthetic domains.

Regarding the transformer-based methods, as shown in Figure 8 and in Table 4 and Table 5, SegFormer-B0 exhibited constant high performance when trained on the synthetic images, comparable to its performance when trained solely on real data. This can be attributed to its lightweight and efficient design, which integrates a simplified hierarchical structure and a minimal decoder, allowing it to generalize well even under limited data conditions and shorter training schedules. On the other hand, Swin-T demonstrated the lowest performance when trained only on synthetic data, even amongst all examined models, potentially attributed to its more complex architecture that relies on shifted window attention and multi-stage hierarchical processing, which, while powerful, has been shown to require larger datasets, longer training schedules, and careful fine-tuning to reach its full potential. This limitation is further exacerbated by the inclusion of the heavy decoder head (UPerNet), which adds additional optimization challenges and increases sensitivity to domain shifts. These characteristics combined with the wide representation span of the images present in the synthetic dataset, as shown in Figure 9, and the relatively small size of the datasets, can justify its lower performance. Conclusively, among the transformer-based models, comparing the F_1_ scores, we have obtained 66.37% over all images better inferences with Swin-T, and 33.63% with SegFormer-B0, when trained with combined real and synthetic data ($D_{r}\cup SD_{filt}$).

Overall, the vast majority of the examined models across both architectural categories exhibit comparable or even superior performance when trained solely on synthetic data compared to when trained exclusively on real data. Furthermore, we observe that all models achieve substantial performance improvements when trained on the combined real and synthetic datasets. These two observations underscore the valuable contribution of synthetic data incorporation in the learning process and highlight the effectiveness and practical utility of the proposed synthetic data generation framework in enhancing model generalization and segmentation performance. Taken together, based on F_1_ score comparisons across all images, U-Net achieved superior segmentation performance in 38% of the cases, followed by Swin-T (23.98%), FCN-ResNet50 (18.1%), SegFormer-B0 (12.97%), and DeepLabV3 (6.94%). These results highlight U-Net as the best-performing model when trained on the combined real and filtered synthetic dataset ($D_{r}\cup SD_{filt}$), as also illustrated in Figure 8.

**Evaluation of synthetic data impact under controlled settings:** In order to further highlight the contribution of synthetic data in the learning process, we conducted an additional experiment using U-Net as the examined model, since, based on the reported results, it is the one that exhibits the largest performance gains in general. In this new experiment, we fixed the total number of training images to match the real-only condition. Specifically, we created a new training configuration composed of 145 real images and 145 synthetic images, totaling 290 samples, the same as in the real-only case ($D_{r}$ in row 3 of Table 3). All training hyperparameters and iteration counts were kept constant across configurations. The results of this experiment, depicted in Table 6, show that this configuration achieves a better performance (1.3% increase in F_1_-score) than the real-only case, indicating that synthetic data can partially replace real data without loss of segmentation accuracy. This provides further evidence that the gains observed in the configuration combining real and synthetic data (last row of Table 3) are not merely due to increased data volume but rather reflect the complementary utility of the synthetic images.

**Table 6:** U-Net performance on the test dataset (FSSD) based on training with randomly selected images 50%, 25%, 25% from the real, synthetic, and semi-synthetic dataset (50% D_*r*_ ∪ 25% SD_*s*_ ∪ 25% SD_*ip*_) in comparison to training with the real dataset only ($D_{r}$). Both training datasets are of the same size. The best-performing values for each metric are highlighted in bold.

Training	Test Metrics				
**Dataset (290 Images)**	**Acc (%)**	**IoU (%)**	**Pr (%)**	**Rec (%)**	**F_1_ (%)**
D_*r*_	81.03	62.62	**68.95**	87.21	77.01
50% D_*r*_ ∪ 25% SD_*s*_ ∪ 25% SD_*ip*_	**81.28**	**64.30**	67.82	**92.53**	**78.27**

### 5.2. Real and Synthetic Dataset Similarity, Role of Prompt Semantics in Dataset Quality

**Real and synthetic data similarity:** To quantitatively assess the distributional similarity between real and synthetic datasets, we computed the Maximum Mean Discrepancy (MMD) between their respective feature representations. Specifically, we extracted deep features using a pre-trained ResNet50 model and computed the MMD between the real dataset and two variants of the synthetic dataset. The resulting scores were ${\mathrm{MMD}}_{D_{r},{\mathrm{SD}}_{s}}=0.0545$ and ${\mathrm{MMD}}_{D_{r},{\mathrm{SD}}_{ip}}=0.0347$. Both scores indicate a reasonable alignment with the real data distribution, as MMD values below 0.1 are typically indicative of good distributional similarity in high-dimensional spaces. Notably, the second synthetic dataset demonstrates significantly closer alignment to the real data, suggesting improvements in generation fidelity—possibly due to enhanced prompt structure, better context grounding, or more effective filtering. These results support the hypothesis that high-quality synthetic data, when properly curated, can closely mimic real-world distributions and serve as a valuable resource for training deep segmentation models.

Additionally, as a second way to assess the similarity between a real-world dataset (D_*r*_) and two synthetic datasets (SD_*s*_ and SD_*ip*_) with a more direct and visual manner, we also used the feature embeddings from each image and applied principal component analysis (PCA) for dimensionality reduction and visualization. The resulting 2D PCA plot, shown in Figure 9, demonstrates a substantial overlap between the three datasets in the feature space (best-fit ellipses overlap), indicating a high degree of visual similarity. Notably, the synthetic datasets exhibit significant alignment with the real-world distribution, suggesting that the synthetic data generation processes effectively capture the global visual characteristics of real imagery. Among them, SD_*ip*_ shows a tighter overlap with D_*r*_, which can be attributed to the ground truth mask prior, while SD_*s*_ exhibits a slightly broader spread, which may imply higher visual diversity or variability in synthesis quality. This kind of deviation may reflect that, while the synthetic dataset captures much of the variance of the real data (hence the overlap), it might also be exploring new areas or patterns in the feature space that are not fully represented in the real data. This is also suggested by the slight deviation in the orientation of the fitted ellipses between the real and synthetic datasets. The novel pattern assumption appears to be verified by the model performance increase when training on the union of the real and synthetic datasets. These findings support the potential utility of synthetic datasets as substitutes or complements to real-world data for training DL models, especially in scenarios where labeled real data is limited or costly to obtain.

**Figure 10:**
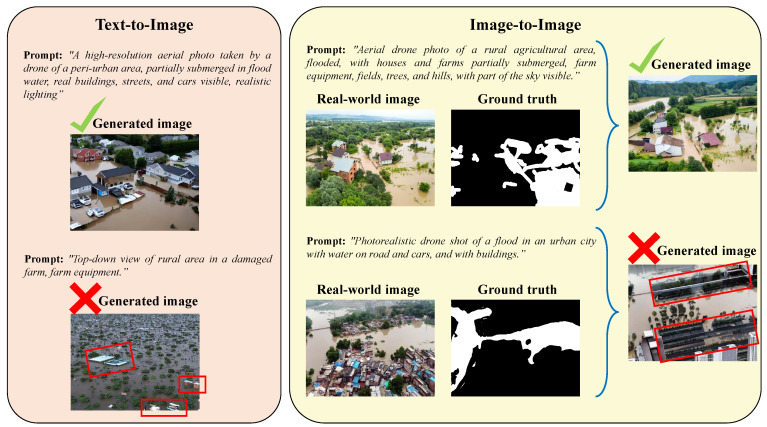
Comparison of accepted (**top**) and rejected (**bottom**) synthetic flood images generated via prompt-to-image (**left**) and image-to image (**right**) translation. Problematic regions—including artifacts, erroneous configurations, and undesired objects—are highlighted with red bounding boxes.

**Prompt structure and semantics in synthetic image quality:** The structure and semantics of textual prompts play a critical role in guiding the fidelity of generated synthetic images. As shown in Figure 10, minor variations in phrasing, specificity, or contextual richness can substantially affect visual realism and alignment with the intended flood scenario. Detailed prompts that include spatial relationships, lighting conditions, environmental context (e.g., urban vs. rural), and object-level cues (e.g., “partially submerged tractors”, “muddy floodwater”, “sky reflections”) consistently result in higher-quality images. Conversely, vague or semantically sparse prompts often lead to artifacts, inconsistencies in water boundaries, or unnatural object placements (e.g., “flooded city” or “flooded river”).

For instance, as illustrated in Figure 10, the use of a vague or underspecified prompt in the text-to-image synthesis pipeline leads to the generation of semantically and structurally inconsistent elements, such as the appearance of building rooftops that were not explicitly requested and exhibit unrealistic geometries (highlighted with red boxes). Similarly, in the image-to-image generation approach conditioned on segmentation masks, employing a more generic prompt results in the synthesis of implausible structures—such as slanted apartment-like buildings—demonstrating that prompt specificity directly impacts the semantic and geometric fidelity of the generated content.

### 5.3. Ablation on Filtering Threshold

As a final task, we investigate the sensitivity of our filtering scheme to the choice of strictness parameter, and we varied the *k*-value in the z-score threshold formulation, presented in Section 3.3. Specifically, we examined k=1.5, k=2, and k=3, corresponding to progressively stricter inclusion criteria for synthetic images based on their distance to real data clusters in feature space. These values were selected to reflect typical thresholds used in outlier detection based on standard deviations from the mean. A threshold of ±3σ standard deviations corresponds to roughly 99.7% coverage under the Gaussian curve, making it a common cutoff for identifying truly extreme values; in our case, outlier synthetic flood images are too dissimilar to real-word ones. Our results, shown in Table 7, demonstrate that a stricter threshold (i.e., k=1.5) consistently yields better segmentation performance across the majority of evaluated models. This suggests that retaining only the most semantically aligned synthetic samples—those with the highest fidelity to real data distributions—is beneficial for generalization.

The performance gains are particularly evident in the convolutional models DeepLabV3 and FCN-ResNet50, and in both transformer-based SegFormer-B0 and Swin-T, which benefit significantly from the more selective filtering. This is likely due to the fact that these architectures possess deep and complex feature extractors, which are more sensitive to the domain discrepancies introduced by low-quality or semantically inconsistent synthetic data. By aggressively filtering out such samples, the learned representations remain more robust and transferable to real-world data. Interestingly, the intermediate threshold k=2 shows the greatest benefit for the U-Net architecture. Unlike the others, U-Net has a more symmetric and shallow encoder–decoder structure, which may allow it to benefit from a slightly larger, more diverse synthetic training set—so long as the noise introduced remains within tolerable bounds. The less favorable performance of the rest of the examined models with k=2 may highlight their greater sensitivity to noisy or out-of-distribution synthetic examples.

**Table 7:** Impact of the z-score threshold parameter *k* on the segmentation performance of different architectures trained on the filtered synthetic dataset. A lower *k* value corresponds to stricter filtering of synthetic images. For each method, the top-performing value per metric is shown in bold.

Method	Thresh. Par. (k)	Filt. Imgs	Test Metrics				
			Acc (%)	IoU (%)	Pr (%)	Rec (%)	F1 (%)
DeepLabV3	-	0/580	79.21	60.94	65.89	89.02	75.73
DeepLabV3	3	3/580	79.11	60.76	65.82	88.77	75.59
DeepLabV3	2	23/580	78.68	59.69	65.73	86.67	74.76
DeepLabV3	1.5	53/580	**79.55**	**61.34**	**66.34**	**89.06**	**76.04**
FCN-ResNet50	-	0/580	83.74	67.82	70.84	94.09	80.83
FCN-ResNet50	3	3/580	83.56	67.64	70.51	94.32	80.70
FCN-ResNet50	2	23/580	82.85	66.90	69.27	**95.14**	80.17
FCN-ResNet50	1.5	53/580	**84.22**	**68.12**	**72.08**	92.54	**81.04**
U-Net	-	0/580	81.91	65.52	68.21	94.32	79.17
U-Net	3	3/580	84.67	68.82	72.67	**92.85**	81.53
U-Net	2	23/580	**87.57**	**72.83**	**78.17**	91.42	**84.28**
U-Net	1.5	53/580	86.97	72.00	76.85	91.94	83.72
SegFormer-B0	-	0/580	80.13	63.61	65.66	95.33	77.76
SegFormer-B0	3	3/580	80.64	64.23	66.26	95.45	78.22
SegFormer-B0	2	23/580	80.92	64.36	66.83	94.57	78.32
SegFormer-B0	1.5	53/580	**81.22**	**64.96**	**66.99**	**95.48**	**78.74**
Swin-T	-	0/580	83.53	66.39	72.11	89.32	79.80
Swin-T	3	3/580	82.12	64.13	70.42	87.78	78.15
Swin-T	2	23/580	81.98	64.28	69.83	89.00	78.26
Swin-T	1.5	53/580	**84.88**	**68.69**	**73.66**	**91.06**	**81.44**

### 5.4. Qualitative Segmentation Results

To evaluate the qualitative performance of the proposed framework, we present segmentation results from the best-performing model, namely U-Net, trained on the combined real-world and filtered synthetic dataset ($D_{r}\cup SD_{filt}$). This configuration consistently achieved the highest F_1_-scores across validation and test sets, confirming its robustness and generalization capability under varying image conditions.

For interpretability and comprehensive assessment, representative segmentation outputs were selected based on the percentile sampling of the F_1_ score distribution over the test set. Specifically, we used the best-performing U-Net model variant, i.e., the variant trained with real and synthetic data ($D_{r}\cup SD_{filt}$), and sorted all segmented test images in descending order of F_1_-scores and extracted five representative cases corresponding to the 0th (best), 20th, 40th, 60th, and 80th percentiles. This approach enables a structured visualization of the model’s performance across different levels of difficulty, from highly accurate segmentations to more challenging scenarios.

**Figure 11:**
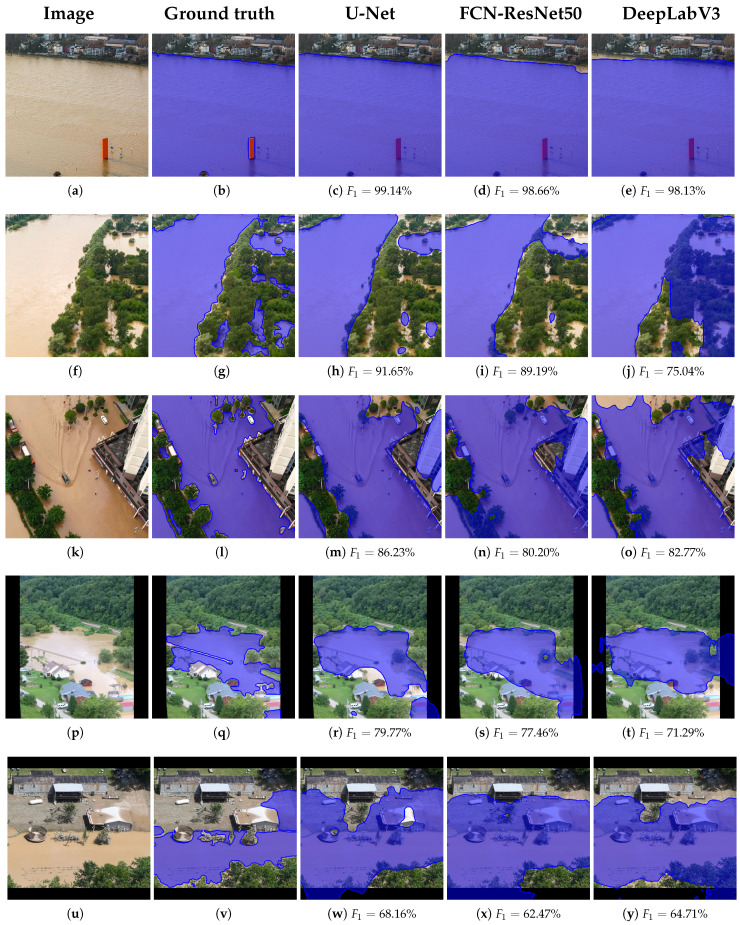
Original images (first column (**a**,**f**,**k**,**p**,**u**)), ground truth masks (second column (**b**,**g**,**l**,**q**,**v**)), and representative segmentation results of the U-Net (third column), FCN-ResNet50 (fourth column), and DeepLabV3 (fifth column) from the FSSD test dataset. The masks are overlaid in blue. Rows correspond to the 0% (best results), 20%, 40%, 60%, and 80% percentile of the descending sorted F_1_-score values of the top-performing CNN architecture, U-Net, trained with real and synthetic data ($D_{r}\cup SD_{filt}$). FCN-ResNet50 and DeepLabV3 were also trained under the same conditions.

As illustrated in Figure 11, the top-ranked examples, e.g., 0th and 20th percentiles (Figure 11c,h), show high-performance segmentation with a near-precise delineation of flood boundaries and minimal false positives or negatives. These cases typically involve well-lit, high-contrast scenes with distinct flood regions and minimal occlusions. In contrast, lower percentile examples, e.g., 60th and 80th percentiles (Figure 11r,w), demonstrate the model’s limitations, often corresponding to visual ambiguities such as flooded vegetation, low contrast between land and water surfaces, or significant reflections and shadow artifacts. Nonetheless, even in these more difficult cases, the U-Net generally preserves the structural integrity of the flooded regions, indicating resilience to challenging input conditions.

These results further substantiate the effectiveness of combining real-world data with selectively filtered synthetic images, which likely enhanced the diversity and coverage of the training set, enabling the U-Net to generalize well across both typical and edge-case scenarios. For comparative reasons, we also present in Figure 11 the corresponding segmentation results of the other two models, FCN-ResNet50 and DeepLabV3, trained with the same configuration as the U-Net, together with the respective F_1_-scores achieved. FCN-ResNet50 performed second best and generally produced better results than the DeepLabV3 architecture, as observed in Figure 11d,i,s, respectively, compared to Figure 11e,j,t.

**Figure 12:**
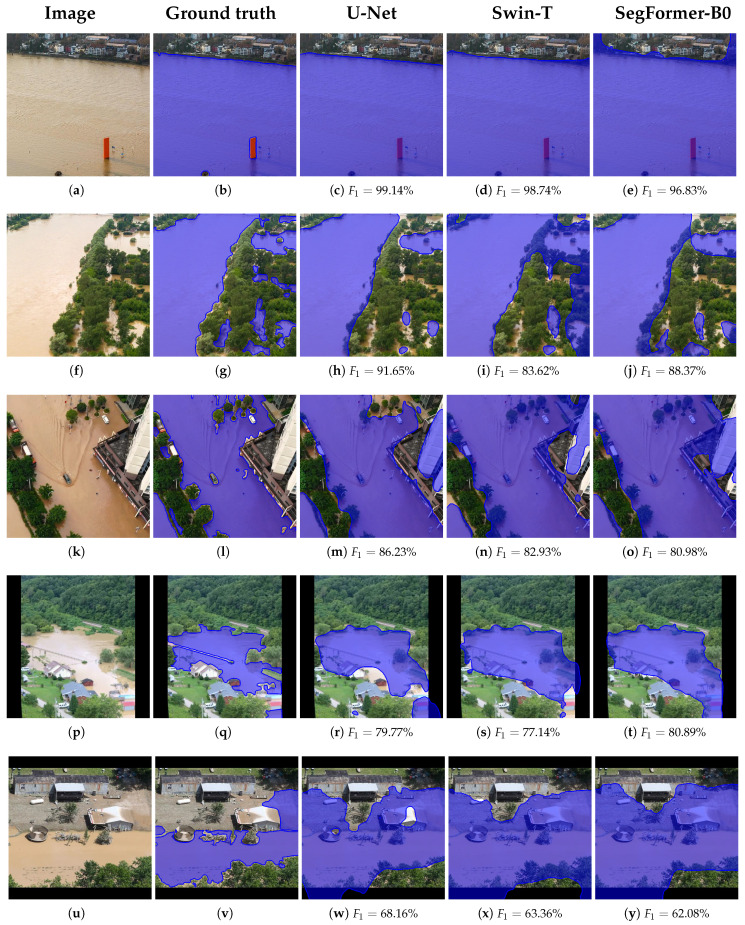
Original images (first column (**a**,**f**,**k**,**p**,**u**)), ground truth masks (second column (**b**,**g**,**l**,**q**,**v**)), and representative segmentation results of the Swin-T (third column), and SegFormer-B0 (fourth column) from the FSSD test dataset. The masks are overlaid in blue. Rows correspond to the 0% (best results), 20%, 40%, 60% and 80% percentile of the descending sorted F_1_-score values of the top-performing overall model, U-Net, trained with real and synthetic data ($D_{r}\cup SD_{filt}$). Swin-T and SegFormer-B0 were also trained under the same conditions.

Similarly, Figure 12 displays the segmentation outcomes of the transformer-based models, Swin-T and SegFormer-B0, trained under identical configurations, alongside their respective F_1_-scores. In comparison to U-Net, both transformer models exhibit challenges in delineating object boundaries, often misclassifying non-flooded regions near flood boundaries as flooded. This is particularly evident in Figure 12i,j,n,o, when contrasted with the more accurate boundary segmentation of U-Net in (h) and (m). This behavior is partly attributed to the architectural characteristics of transformer models, such as the use of token-based representations and the shifted window mechanism in Swin-T, which can reduce spatial precision and impair the capture of fine-grained boundary details. Additionally, unlike U-Net, which incorporates strong inductive biases through convolutional operations and skip connections, transformer models lack inherent spatial locality and are more dependent on large-scale data to generalize effectively. However, there are cases such as Figure 12d,e,t, where transformer-based methods perform comparably to convolution-based ones, indicating the challenging nature of the task and the absence of a method that generalizes in every case. Overall, U-Net demonstrated superior detail preservation, which may be attributed to its stronger generalization capability under limited data conditions, whereas transformer-based architectures typically benefit from larger training sets and extended training time.

**Figure 13:**
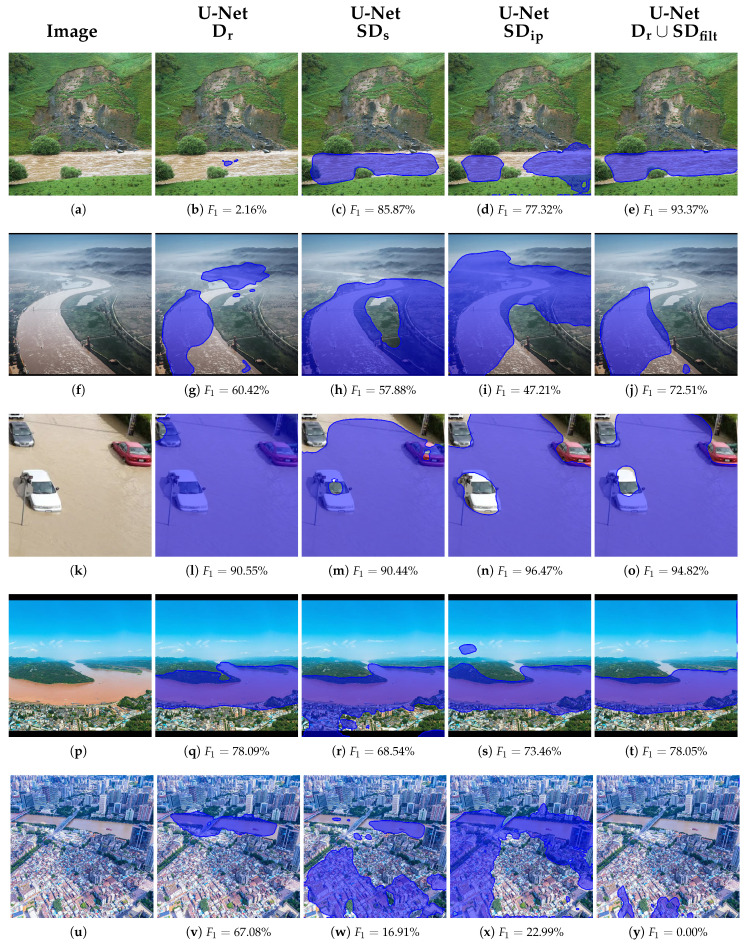
Original images (first column (**a**,**f**,**k**,**p**,**u**)) and representative segmentation results from the FSSD test dataset of the U-Net architecture trained with different datasets: real-world images and ground truths (second column), synthetic images and corresponding pseudo-label masks (third column), semi-synthetic images and corresponding pseudo-label masks (fourth column), and combined real-world data and filtered synthetic datasets and corresponding ground truth and pseudo-labels (fifth column). Segmentations are overlaid in blue. Rows correspond to the 0% (best results), 25%, 50%, 75% and 100% (worst results) percentile of the descending sorted F_1_-score differences of the top performing U-Net architecture (last columns) and its counterpart trained with real-world images and ground truth (second column).

In Figure 13, we showcase the effectiveness of synthetic and semi-synthetic data for flood segmentation, presenting representative segmentation results from the same U-Net architecture trained under four distinct configurations: (i) using real-world images with manually annotated ground truth masks, (ii) using purely synthetic images generated via Stable Diffusion with automatically produced pseudo-labels, (iii) using semi-synthetic images created through image inpainting with corresponding pseudo-labels, and (iv) a combined dataset comprising real, synthetic, and semi-synthetic samples with their respective ground truth and pseudo-label masks. Five representative cases were chosen among the descending sorted differences of the F_1_-scores of the best- and-worst performing configurations, (iv) and (i), corresponding to the highest difference (first row), the differences of the 25th, 50th, 75th percentiles, and the lowest difference (last row).

Among the four cases, the combined dataset (case iv in the last column) achieved the highest performance in terms of segmentation accuracy, as measured by the F_1_-score. This indicates that the integration of real-world examples with synthetic and semi-synthetic data can significantly enhance the model’s generalization capability. The observed improvement is attributed to the increased variability and diversity introduced by the synthetic samples, which augment the training distribution and expose the model to a wider range of environmental conditions, textures, and structural layouts. This diversity appears to regularize the training and prevent overfitting to the limited real-world data.

Interestingly, both synthetic (case ii, third column) and semi-synthetic (case iii, fourth column) training independently produced segmentation results that were not only comparable to, but in some cases exceeded, the performance of models trained solely on real-world data (case i, second column), as observed in Figure 13c,d,n compared to Figure 13b,l. This highlights the capacity of U-Net to learn robust features even when trained exclusively on artificially generated data, provided that the pseudo-labels contain a sufficient region of interest despite their inherent noise. It also underscores the model’s resilience to imperfect supervision and demonstrates the potential of unsupervised or weakly supervised learning approaches for remote sensing tasks where high-quality annotated datasets are often scarce. The synthetic data, generated entirely from random noise using a generative diffusion process, provided a broad distribution of flood-like appearances, while the semi-synthetic data retained structural realism from the real imagery due to inpainting guided by true segmentation masks. Of course, there are also failure cases where complex patterns could not be learned and synthetic generated data as well as their combination with real data seemingly confused the model (see Figure 13w–y).

These findings collectively suggest that, in the absence of annotated datasets, the use of synthetic or semi-synthetic imagery in conjunction with automatic pseudo-labeling can offer a viable alternative for training deep segmentation models. Furthermore, combining such data with limited real-world samples results in a synergistic effect that further improves segmentation accuracy, advocating for hybrid training strategies in future work.

## 6. Conclusions

In this work, we presented a framework for constructing a synthetic dataset aimed at the task of flood segmentation in UAV imagery. Our approach integrates two distinct generative strategies: a text-to-image synthesis pipeline guided by flood-related textual prompts, and an image-to-image translation paradigm that leverages real segmentation masks as structural priors to generate realistic flood scenes via inpainting. These complementary approaches are unified to produce a diverse and semantically meaningful dataset.

To address the challenge of missing ground truth annotations for segmentation, we employ an unsupervised pseudo-labeling strategy to generate segmentation masks for the synthetic images. This allows us to construct paired image–mask samples without the need for manual annotation, significantly reducing the cost and effort typically associated with dataset curation. Furthermore, we incorporate a filtering stage based on outlier detection to ensure the realism and structural fidelity of the generated images, discarding samples that do not meet quality standards. To evaluate the effectiveness of the synthetic dataset, we conducted experiments with five top-performing flood segmentation models, three CNN and two transformer-based, assessing their performance on a real-world benchmark dataset. Our findings demonstrate that training with the synthetic data, even when annotated via unsupervised pseudo-labeling, not only leads to minor model performance drops, but the combination of synthetic and real-world data during training is able to improve model generalization and robustness. Our framework offers a scalable and low-cost solution for generating annotated flood segmentation datasets, with practical applications in disaster monitoring, remote sensing, and other vision-based environmental analysis tasks.

While our current framework provides a scalable solution for generating synthetic flood segmentation datasets, several promising directions remain open for future exploration. These include improving the accuracy of the unsupervised pseudo-labeling method by integrating stronger segmentation priors or combining multiple weak supervision signals. We also plan to explore domain adaptation techniques—such as adversarial training and feature alignment—to further reduce the gap between synthetic and real data. Lastly, we aim to extend the framework by considering multi-modal data synthesis for broader environmental monitoring applications.

## Data Availability

The original contributions presented in this study are included in the article; further inquiries or requests can be directed to the corresponding authors.

## Abbreviations

The following abbreviations are used in this manuscript:

ADNet	Attentive Decoder Network
AI	Artificial Intelligence
ASPP	Atrous Spatial Pyramid Pooling
BiT	Bitemporal image Transformer
CNN	Convolutional Neural Network
CV	Computer Vision
DELTA	Deep Earth Learning, Tools, and Analysis
DL	Deep Learning
DNN	Deep Neural Network
ENet	Efficient Neural Network
FAD	Flood Area Dataset
FCN	Fully Convolutional Network
FSSD	Flood Semantic Segmentation Dataset
GAN	Generative Adversarial Network
IoU	Intersection over Union
ML	Machine Learning
MMD	Maximum Mean Discrepancy
MRF	Markov Random Field
NDWI	Normalized Difference Water Index
PCA	Principal Component Analysis
PR	Pattern Recognition
ResNet	Residual Network
SAM	Segment Anything Model
SAR	Synthetic Aperture Radar
SegFormer	Segmentation Transformer
SSSS	Semi-Supervised Semantic Segmentation
UAV	Unmanned Aerial Vehicle
UPerNet	Unified Perceptual Parsing Network
VAE	Variational Autoencoder
WSSS	Weakly Supervised Semantic Segmentation
